# Development of Quasi-Passive Back-Support Exoskeleton with Compact Variable Gravity Compensation Module and Bio-Inspired Hip Joint Mechanism

**DOI:** 10.3390/biomimetics9030173

**Published:** 2024-03-13

**Authors:** Gijoon Song, Junyoung Moon, Jehyeok Kim, Giuk Lee

**Affiliations:** 1 School of Mechanical Engineering, Chung-Ang University, Seoul 06974, Republic of Korea; robo3017@cau.ac.kr (G.S.); mjyoung5@cau.ac.kr (J.M.); 2 Department of Mechanical Engineering, Université Laval, Québec, QC G1V 0A6, Canada; michael.jh.kim0225@gmail.com

**Keywords:** wearable robots, back support, quasi-passive mechanism, variable assistance

## Abstract

The back support exoskeletons have garnered significant attention to alleviate musculoskeletal injuries, prevalent in industrial settings. In this paper, we propose AeBS, a quasi-passive back-support exoskeleton developed to provide variable assistive torque across the entire range of hip joint motion, for tasks with frequent load changes. AeBS can adjust the assistive torque levels while minimizing energy for the torque variation without constraining the range of motion of the hip joint. To match the requisite assistance levels for back support, a compact variable gravity compensation module with reinforced elastic elements is applied to AeBS. Additionally, we devised a bio-inspired hip joint mechanism that mimics the configuration of the human hip axis to ensure the free body motion of the wearer, significantly affecting assistive torque transmission and wearing comfort. Benchtop testing showed that AeBS has a variable assistive torque range of 5.81 Nm (ranging from 1.23 to 7.04 Nm) across a targeted hip flexion range of 135°. Furthermore, a questionnaire survey revealed that the bio-inspired hip joint mechanism effectively facilitates the transmission of the intended assistive torque while enhancing wearer comfort.

## 1. Introduction

Low back injuries have been problems continuously in industrial workplace for decades. Repetitive manual handling leads to muscle fatigue in the back muscles, resulting in low back injuries [1,2,3]. Moreover, ligament damage and injuries caused by low back muscle fatigue and low back injury contribute to low back pain [4]. Low back injuries take half of low back-related musculoskeletal disorders [5]. As a result of those injuries, the quality of life of workers decreased [6], as well as causing social issues. The costs associated with back pain have continuously risen, and are expected to continue increasing in the coming decades [7]. Overall, low back pain caused by manual material handling significantly limits the physical activity of workers and generates broader societal concerns.

To address these concerns, back-support exoskeletons have been developed, which can reduce the burden on the lower back to prevent low back injuries. Many researchers have developed and verified the effect of various back support exoskeletons, which can be divided into two types of assistive force generating devices: active and passive. Active type back-support exoskeletons use active components like powered actuators as a source of assistive force, such as electric motors [8,9]. On the contrary, passive type back-support exoskeletons use passive components, such as coil or gas springs [10,11], to generate assistive force. For example, H-Wex and XoTrunk, which are active back-support exoskeletons using BLDC motors, can support manual material lifting tasks by providing assistive torque to the hip joint [12,13]. Laevo, a passive back-support exoskeleton, can assist manual material lifting tasks by providing assistive force through a thigh cuff using a gas spring and cam [14]. SPEXOR, another passive back-support exoskeleton, assists in the sagittal plane motion of manual material lifting by providing assistive torque to both the hip joint and the L5S1 joint, using a coil spring system and flexible carbon beam [15,16].

However, to effectively reduce the risk of lower back injury, the exoskeleton must be able to provide variable magnitude of assistive torque to the human body. This variability is essential because the moment applied to the human body during payload handling varies [17,18]. Moreover, the working environment can influence the moment applied to the human body. Actual industrial sites represent complex working environments, where various tasks are discontinuously performed. For example, various weight payloads may be handled in a random order. Therefore, a back-support exoskeleton that can swiftly and easily adjust the assistive torque must be developed. Traditional active-type exoskeletons can freely adjust the magnitude of assistive torque. However, their significant energy consumption limits prolonged usage [19]. In contrast, passive-type exoskeletons do not have operating time limitations, as they do not require energy. However, the adjustment of assistive torque is difficult. The previously reported passive-type exoskeletons need to adjust the pre-deformation of elastic components either manually [15] or using another version of robots [20,21]. Because of these limitations, it is challenging to adjust the magnitude of assistive torque quickly during operation.

To address these problems, the concept of quasi-passive exoskeletons with variable assistive torque and low energy consumption has been suggested. These quasi-passive exoskeletons use a passive element as the source of the assistive force. Moreover, they have the function of being able to adjust the characteristics of the assistive force, such as magnitude, by using a small actuator [19,22,23]. This characteristic allows quasi-passive exoskeletons to operate with low energy consumption while taking advantage of the long operating time of passive exoskeletons and the easy variability of the assistive force magnitude of active exoskeletons. A representative example of a quasi-passive exoskeleton is MRLift, which uses an MR (Magnetorheological) fluid and a coil spring to adjust braking force by applying current to the MR fluid [24,25]. Another quasi-passive exoskeleton prototype can distinguish between squat, stoop, and left or right stoop motions by recognizing user intent, thereby determining the engagement timing of assistive torque [23]. This prototype can also adjust the magnitude of assistive torque for each leg by modifying precompression of the coil spring, which is the source of assistive torque, enabling independent control of the torque of both legs. These quasi-passive exoskeletons successfully verified the effect of those functions. However, MRLift continuously consumes energy to maintain the adjusted braking force, potentially limiting its operating time in industrial workplaces. The other reported prototype presents limitations related to the range-of-motion with variation in the assistive torque, and also there’s another limitation which the precompression of the coil spring must be manually adjusted. Because the prototype uses the precompression of the coil spring to able variable assist function, range-of-motion limitation occurs. Also the precompression of the spring must be manually adjusted, which can be an obstacle to use in industrial sites.

To mitigate the limitations of existing quasi-passive exoskeletons and provide proper adjustable assistive torque to the human body, we developed a quasi-passive exoskeleton named the AD exo-Back Support (AeBS). By using the compact variable gravity compensation (CVGC-II) mechanism [26], AeBS can provide assistive torque of various magnitudes to the human body without restricting the range-of-motion. The lever mechanism of CVGC-II eliminates the need for precompression of passive elements, thereby ensuring that the range-of-motion is not limited. Additionally, the use of a fully passive mechanism eliminates the need for continuous energy to maintain the adjusted assistive torque. Furthermore, the hip joint structure which is inspired by the human hip joint axis configuration ensures free body motion and secure connection between the robot and the human body. Consequently, AeBS can adjust assistive torque without limiting human motion variability while operating for extended periods.

The remaining paper is structured as follows. Section 2 introduces the hardware design concept of the novel quasi-passive exoskeleton, AeBS. Section 3 describes the hardware evaluation of AeBS to validate the variable assist function. Section 4 presents the evaluation results. Section 5 presents the concluding remarks.

## 2. Development of Quasi-Passive Back-Support Exoskeleton: AeBS

### 2.1. General Configuration and Specification of Robot

In the development of a back-support exoskeleton, two key aspects must be considered to provide appropriate assistive torque in complex working environments. First, the assistive torque must be appropriately varied to adapt to various moments exerted on the human body. Second, the exoskeleton must ensure unrestricted motion of the human body during manual material handling tasks in complex working environments.

**Figure 1:**
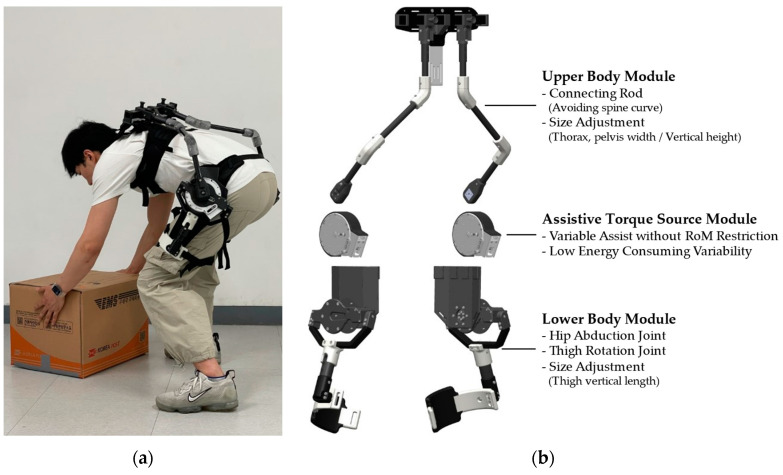
(**a**) The actual image of AeBS; (**b**) three operating modules of AeBS.

The novel quasi-passive back-support exoskeleton AeBS was developed under these considerations. Figure 1a shows the overall appearance of AeBS. As shown in Figure 1b, AeBS consists of three operating modules: the upper body module, lower body module, and assistive torque source module. The upper and lower body modules serve as fixed connection points between the human body and exoskeleton while transmitting assistive torque. Additionally, these modules also have elements to ensure the free motion of the human body, such as collision-avoiding connecting rods, hip abduction joints, and thigh cuff rotation. These functions make effective assistive torque transfer to the human body by allowing free motion of the human body. The assistive torque source module is the modified CVGC-II, which is a modified version of CVGC-II [26] tailored for hip joint assistance. The variable gravity compensation mechanism of the CVGC-II system can change the magnitude of the assistive torque by adjusting the leverage ratio through moving the pivot location. Additionally, because this assistive torque adjustment is unrelated to the precompression of the spring system, there are no restrictions on the range of motion that the CVGC-II assist system can provide. Accordingly, each characteristic of the variable gravity compensation mechanism is suitable for realizing variable assistive torque and unrestricted motion of the human body using a back-support exoskeleton. The modified CVGC-II provides assistive torque to the hip joint, contributing to variable assistive torque function and free motion-ensuring function.

AeBS has a total weight of approximately 5 kg, with the modified CVGC-II systems accounting for 2.36 kg. The structures of the upper and lower body modules are constructed using 3D-printed parts, carbon plates, and carbon pipes to reduce weight and simplify the overall structure. The comparison of the weight of the robots is shown in Table 1.

### 2.2. Generation of Assistive Torque

**Figure 2:**
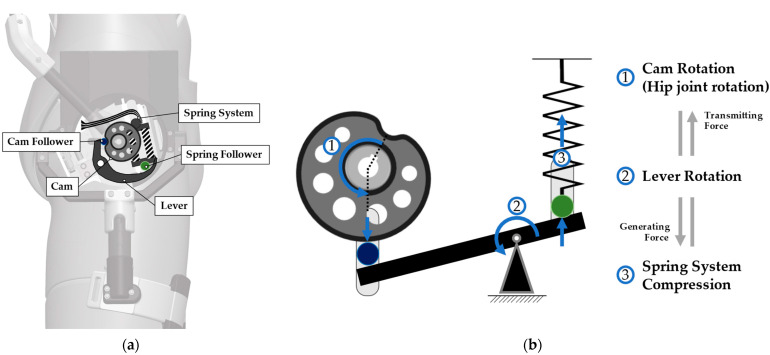
(**a**) Key components of CVGC-II system for generating assistive torque; (**b**) geometric relationship between key components. The spring system is compressed by the rotation of the cam connected to the upper body module.

The CVGC-II device is designed to generate assistive torque. By aligning the torque output joint with the hip joint, assistive torque can be provided to the hip joint, which is one of the major working joints during manual material lifting/lowering. As shown in Figure 2a, the torque output axis is connected to the upper body module, and the main frame of CVGC-II system is connected to the lower body module. Therefore, the CVGC-II system can rotate along with the hip joint rotation.

The components of the CVGC-II system are shown in Figure 2a. CVGC-II system majorly consists of a cam, lever, and spring system [27], and each component interacts through the cam follower and spring follower. Figure 2b illustrates the geometric relationship and relative movement between each component. The torque output axis, connected to the upper body module, is connected to the cam. Thus, the movement of upper body is converted to cam rotation. When the cam rotates, the lever which is contacted to the cam also rotates. The lever movement results in the displacement of the spring system. The reaction force induced by the spring displacement generates a normal force on the cam surface. Because the contact angle at the cam surface is not zero, the surface normal force generates an assistive torque along the cam axis.

To enhance the effectiveness of hip joint assistance using the CVGC-II system, we replaced the coil springs of the CVGC-II system to increase the maximum assistive torque. The original CVGC-II system was designed for 180° rotation. However, AeBS requires only a limited range-of-motion of the hip joint during material lifting/lowering tasks. The squat, a common material lifting/lowering movement, typically involves a maximum hip flexion range of approximately 120° [28,29]. Thus, we modified the coil spring (SWL16-40, MISUMI, Tokyo, Japan) to ensure that the maximum compression displacement of the spring occurs at 135°.

### 2.3. Variability of Assistive Torque without Range-of-Motion Restriction

Adjustable assistance is a key function of back-support exoskeletons. AeBS can adjust the magnitude of assistive torque through the variable pivot mechanism of the modified CVGC-II system. The reaction force from the spring system displacement is transmitted through the lever. The normal force finally transmitted to the cam varies with the leverage ratio. This leverage ratio depends on the pivot position, which can be adjusted by the screw mechanism of the modified CVGC-II.

**Figure 3:**
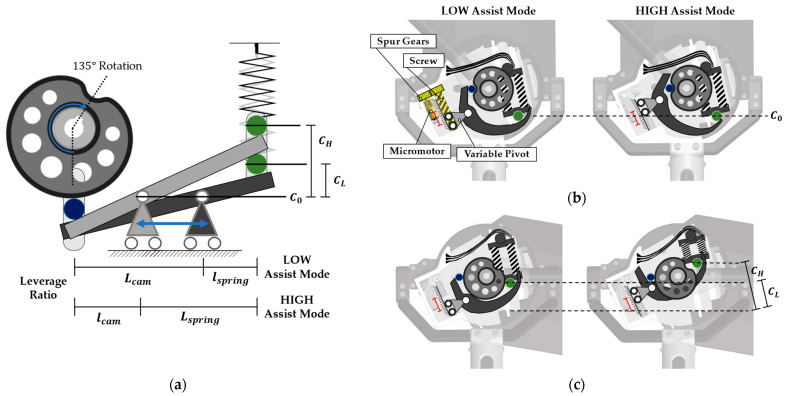
(**a**) Change in leverage ratio with varying pivot positions; (**b**) despite changes in the pivot position, the initial position of the spring follower remains identical. Thus, changing the assistive mode does not affect the hip joint range-of-motion; (**c**) changing the leverage ratio varies the compression length of the spring system, enabling adjustment of the assistive torque.

Figure 3a shows the change in the leverage ratio and spring system displacement between LOW and HIGH assist modes through pivot position adjustment. The leverage ratio in the LOW assist mode is determined as follows:

(1) $$Leverage\;Ratio=\frac{l_{spring}}{L_{cam}}\;$$

When $l_{spring}$ increases to $L_{spring}$ and $L_{cam}$ decreases to $l_{cam}$ through pivot position adjustment, the amplification ratio of the reaction force associated with spring displacement increases. Consequently, the normal force on the cam surface increases, leading to a corresponding increase in the output assistive torque.

Furthermore, a change in the pivot position drives a change in spring system displacement. When the pivot position changes, a different amount of displacement occurs on the spring system despite the same cam profile and rotation angle. When $l_{spring}$ increases to $L_{spring}\;$ and $L_{cam}$ decreases to $l_{cam}$, the travel distance of the spring follower increases from $C_{L}$ to $C_{H}$, starting from the initial position $C_{0}$. Thus, the maximum displacement of the spring system increases. This increased spring system displacement leads to a larger normal force on the cam surface, and the output assistive torque increases in accordance.

As shown in Figure 3b, even when the pivot position changes, there is no precompression of the spring system due to the design of the shape of the lever. Additionally, the modified CVGC-II system can use the complete 135° rotation range, even when the maximum spring system displacement changes. In other words, even with variability in assistive torque, AeBS does not impose any range-of-motion restriction on the hip joint.

The position of the pivot in the system can be automatically adjusted. As shown in Figure 3b, a screw mechanism was used to make the linear movement of the pivot position. A micromotor is used to automatically rotate the pivot screw axis. Thus, the wearer can easily change the magnitude of assistive torque using the switches that control the position of the pivot by rotating the micromotor. During the pivot adjustment process, the energy consumption of the robot is low because the power consumption of the micromotor is only 1 W. The micromotor only operates and consumes energy when adjusting the assistive torque. This is possible because of the self-locking characteristic of the transfer screw that changes the pivot location of the lever, eliminating the need for continuous energy consumption to maintain the adjusted value of the assistive torque. Furthermore, due to its high speed, this assistive torque adjustment system using a micromotor is suitable for active change of assistive strategies in complex working environments. Specifically, the max–min torque modes can be changed within 0.9 s.

### 2.4. Robot Structure for Allowing Free Body Motion

**Figure 4:**
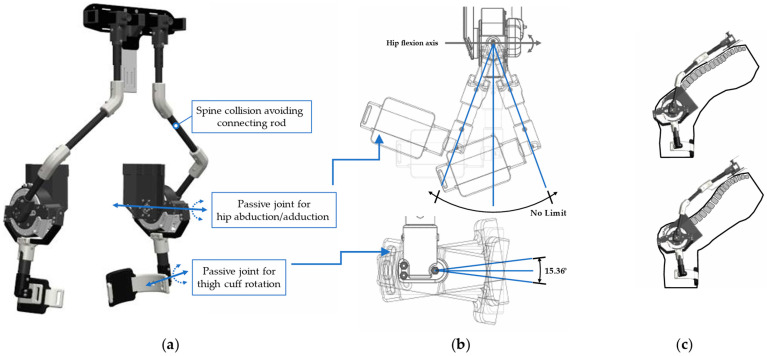
(**a**) AeBS functions enabling various movements; (**b**) range of motion of hip abduction joint and thigh rotation joint; (**c**) the connecting rod avoiding collision with the human spine. The connecting rod can avoid various curve shapes of the upper body.

The ability of a robot to effectively follow the body movement is also a key function to ensure free motion. Figure 4a shows the functions that allow AeBS to facilitate various human movements without interference. Because AeBS uses a thigh cuff and an upper back plate as a support to provide assistive torque, the interface between the human body and the robot must securely attach without hindering human body movement. Additionally, because AeBS is basically an exoskeleton, its structure should not collide with a human body segment or hinder its motion.

A hip abduction joint can be a way to ensure free motion. Hip abduction is critical for exoskeletons. Hip abduction motion is essential for making a stable posture by adjusting the step width according to the payload size and shape and workplace environment during material lifting/lowering. This motion is essential not only for manual lifting/lowering, but also for stable normal working. Thus, a hip abduction joint was added in AeBS, allowing it to follow human hip abduction motion. At this time, free motion can be ensured only when the hip abduction movement and hip flexion movement work organically without interfering with each other. Therefore, we designed the hip abduction rotation axis to intersect the hip flexion axis, just as the human hip joint is a ball joint, and these two axes intersect. The mechanism of the hip abduction joint is shown in Figure 4b.

Similarly, a thigh rotation joint also can be a way to ensure free motion. The thigh cuff of the robot should be attached securely and comfortably to the leg because it acts as a support fixture when delivering assistive torque to the hip joint. To make secure and comfortable attachment, it must be able to flexibly adapt to various thigh thickness and tapered lines that change accordingly. Thus, we added a thigh rotation joint to ensure that the thigh cuff could rotate to adapt to the tapered line of the thigh and thus be in close contact with the thigh. The working mechanism and range-of-motion of the thigh rotation joint are illustrated in Figure 4b.

The connecting rod that avoids collision with the spine is essential for free motion. The spine curve that occurs during material lifting movements such as squatting and stooping is one of the reasons that makes it difficult to design the connecting rod between the thigh and upper back-support points of the exoskeleton. If the spine curvature is not considered when designing the structure of the exoskeleton, a collision could occur between the exoskeleton and the human body, which could restrict movement or cause an injury. However, a complex mechanism is required to design a structure that can fully follow the spine curve motion, which can increase the system complexity and increase the robot weight. Thus, we designed a connecting rod that can avoid the spinal curve. Considering the position of each vertebra during a stoop posture, a widely used lifting technique characterized by significant spine curve, a connecting rod was designed to three-dimensionally avoid positions with large lumbar bending. This design enabled the connection of the upper and lower body modules without additional structures. The connecting rod used carbon pipes to maintain a low weight and transmit assistive torque with minimum loss, and 3D-printed parts were used for the pipe coupler at the rod-bending section. The shape of the connecting rod, designed to avoid collisions with the human body, is shown in Figure 4c.

The left/right independent controllability of assistive torque can also be a way to free motion. The assistive torque required in the left/right body segment may differ across various human postures during tasks in complex working environments. Providing improper assistive torque to the left/right body may limit and hinder the intent of the wearer. Thus, we used two modified CVGC-II systems in AeBS, placed on the left and right sides of the pelvis, enabling independent control of the assistive torque provided to the left/right hip joint. The overall composition, function, and movement of the robot covered in Section 2 can be found in Video S1.

## 3. Hardware Evaluation

### 3.1. Assistive Torque Transmission Performance Evaluation of the Robot Structure

#### 3.1.1. Test Bench and Experimental Protocol

AeBS has multiple functions, including extra joints to ensure unrestricted human motion. The robot structure is composed of carbon pipes, carbon plates, and 3D printed parts such as ABS. Because of the characteristics of these materials, which can easily deform elastically, the assistive torque transmission performance of the robot structure should be validated.

**Figure 5:**
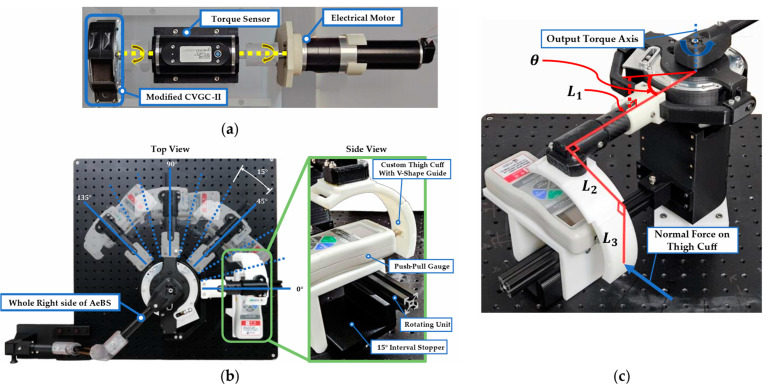
(**a**) Test bench setups for measuring the torque profile of modified CVGC-II, the assistive torque source module of AeBS. The rotary torque sensor was used to measure the torque profile of modified CVGC-II, and the electrical motor was used to rotate the modified CVGC-II torque output axis; (**b**) test bench setup for measuring the torque profile through the robot structure. The whole right side of the robot was attached, and the normal force on the thigh cuff was measured in 15 degrees intervals; (**c**) geometry of the robot structure to calculate the assistive torque from the normal force on the thigh cuff.

To evaluate the transmitting performance of the robot structure, two test benches were used. The first test bench was used to measure the original torque profile of the assistive source device, modified CVGC-II, as shown in Figure 5a. The test bench consisted of the modified CVGC-II, an electrical motor (RE50, Maxon, Sachseln, Switzerland), and a rotary torque sensor (M425, datum electronics, Isle of Wight, United Kingdom). One side of the rotary torque sensor was connected with the torque output axis of the modified CVGC-II through Oldham coupling. The other side of the torque sensor was connected with the rotation axis of the electrical motor through Oldham coupling. To accurately rotate the complete system, a real-time motor controller (Gold solo twitter, Elmo Motion Control, Petah Tikva, Israel) was used along with PC software (Elmo Application Studio II 2.4.0.0, Elmo Motion Control, Petah Tikva, Israel).

The assistive torque was measured under conditions mimicking human motion during manual material handling. The DOWN phase, representing the downward movement of the human upper body to pick up or drop a payload, was measured with motor axis rotation from 0° to 135° in 15° intervals. The UP phase, representing the upward movement of the upper body, was measured with motor rotation from 135° to 0°, in the reverse direction of the DOWN phase. In addition, two modes of assistive torque magnitude were considered in the measurement: the HIGH assist mode, with the maximum leverage ratio, and the LOW assist mode, with the minimum leverage ratio.

The second test bench was used to measure the transmitted torque profile through the entire robot structure, as shown in Figure 5b. This test bench included the whole right side of the robot, anchors for fixing the robot structure, and a rotating unit to measure the normal force on the thigh cuff. The upper-body fixing point was attached to the test bench using an anchor. The rotating unit rotated the hip joint along the hip joint by pushing the thigh cuff. The contact point between the thigh cuff and rotating unit used a push–pull gauge (DS2-500N, OPTECH, Shanghai, China) to measure the normal force on the thigh cuff surface. The hip joint axis rotation was conducted from 0° to 135° in 15° intervals. To ensure measurement reliability, this process was repeated five times. To ensure the secure and precise connection between the push–pull gauge and thigh cuff, a custom thigh cuff was used, featuring a V-shape guide directing the tip of the push–pull gauge to the center of the thigh cuff.

#### 3.1.2. Assistive Torque Calculation Based on the Structure Geometry

To calculate the assistive torque from the normal force on the thigh cuff, a formula was induced based on the robot geometry. The geometric measurement of the robot structure is shown in Figure 5c. Assuming the structure of the robot to be a rigid body and forces applied in a static situation, $L_{2}$ does not affect the torque calculation. If θ represents the hip abduction angle and F denotes the normal force on the thigh cuff, the assistive torque can be calculated using the normal force on the thigh cuff is as follows:

(2) $$Assistive\;Torque=\left(L_{1}\cos\theta +L_{3}\sin\theta \right)\times F$$

In this experiment, the hip abduction joint was set as 0° to eliminate the influence of the weight of the lower body module. The actual measured length $L_{1}$ was 204 mm. Therefore, the assistive torque profile that the human body actually received through the robot structure could be calculated.

#### 3.1.3. Torque Transmission Performance Evaluation Result

**Figure 6:**
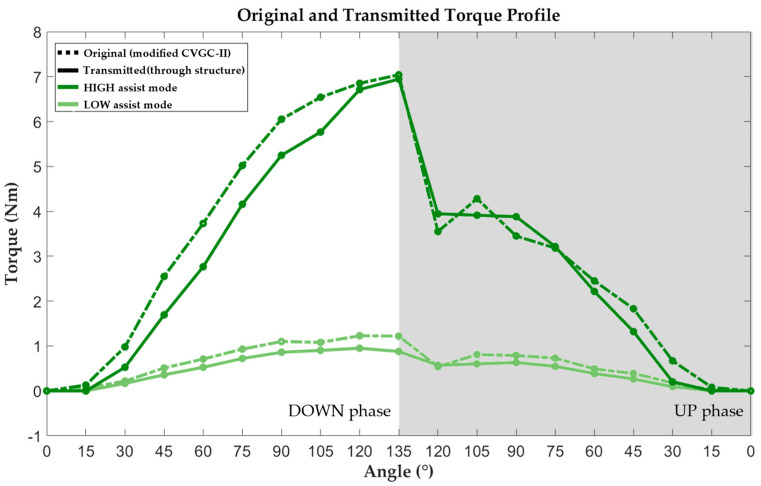
Measured original and transmitted assistive torque profiles in LOW and HIGH assist modes. “Original” refers to the torque profile of the modified CVGC-II system. “Transmitted” refers to the torque profile of the modified CVGC-II system transmitted through the robot structure.

The measured original and transmitted assistive torque results are shown in Figure 6. At 0° and 15° of hip joint rotation during the measurement of the transmitted assistive torque, no contact occurred between the thigh cuff and push–pull gauge. Thus, the value of the assistive torque at 0° and 15° rotation was considered zero. For the transmitted torque profile, the mean value of five repetitions was used. The results indicated that the root-mean-square (RMS) errors between the original and transmitted assistive torque values were 0.51 and 0.17 Nm for the HIGH and LOW assist modes, respectively. A comparison of the original and transmitted torque profiles revealed that the maximum torque value and the tendency of the magnitude of the assistive torque were similar throughout the DOWN and UP phases. The hysteresis of the system was also measured in both original and transmitted torque profiles. In the UP phase, the assistive torque profile was down-shifted compared with that in the DOWN phase in both assist mode conditions.

In addition, because of the modified coil spring, the maximum assistive torque of the modified CVGC-II system in the DOWN phase increased to 7.04 Nm, relative to the maximum value of 5.1 Nm of CVGC-II. Additionally, the variable range of assistive torque extended from 4.1 Nm to 5.81 Nm [26].Therefore, CVGC-II was successfully modified to assist the hip joint with higher torque within a wider range.

### 3.2. Usability Evaluation Using Questionnaire

#### 3.2.1. Experimental Protocol

**Figure 7:**
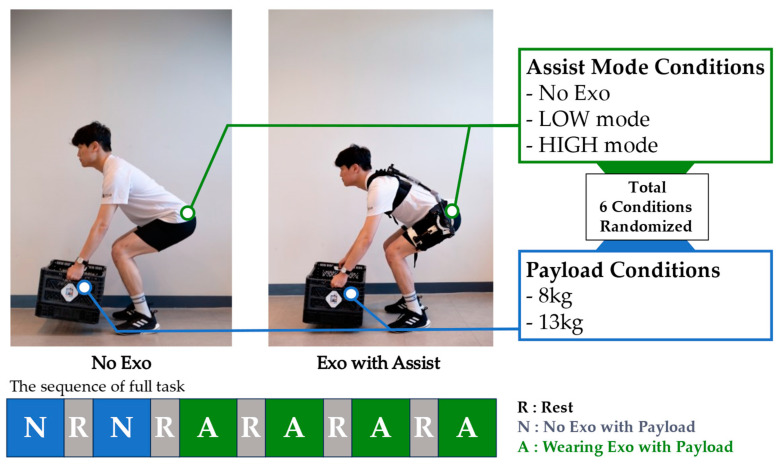
Usability test conditions based on different assist modes and payload conditions. The task sequence was randomized, and a rest session was implemented between each condition.

A questionnaire was used to evaluate the usability of the AeBS, including its variable assist function. The experimental protocol was approved by the Chung-Ang University Institutional Review Board (approval number 1041078-202107-HR-214-01C), and all procedures were conducted in accordance with the approved study protocol. Seven male participants (age: 26±2 years, height: 169.9±4 cm, and weight: 64.9±4.6 kg) answered to the questionnaire after completing material lifting/lowering tasks in a squat posture. The sequence of the task with six conditions is shown in Figure 7, including two payload weight options and three assistive torque options based on exoskeleton-wearing dependency. The three assistive torque options were No Exo (not wearing exoskeleton), LOW (wearing exoskeleton with LOW assist mode), and HIGH (wearing exoskeleton with HIGH assist mode). The two assist modes were selected under consideration of practical usage. The LOW assist mode provides the lowest assistive torque, which can be used in lightweight material lifting/lowering tasks. In contrast, the HIGH mode provides the highest assistive torque, which can be used with heavy material lifting/lowering tasks. The two payload options were 8 kg and 13 kg, including the box. The task conditions were randomized to imitate a complex working environment and eliminate the order effect. However, the No Exo condition was conducted first to facilitate a comparison of the results in the presence and absence of the exoskeleton. The participants were allowed to rest for about 5 min between each task condition, and they selected the pace of lifting/lowering based on their comfort. In a single condition of the experimental task, the participants performed 13 repetitions of lifting/lowering tasks.

The questionnaire consists of the following seven questions for evaluating the usability, such as the robot weight, comfort, and variability of assistive torque. There were five levels of response in the questionnaire, and subject freely chose between each level to respond [12,30].

#### 3.2.2. Usability Evaluation Results

Table 2 lists the questions and response scores of the usability evaluation. The responses for Q1 and Q2 indicated that the weight of the robot was moderate, and users did not experience discomfort while wearing the robot. The average responses for Q3 to Q6 were consistently above four out of five across all participants. The responses for Q3 and Q4 indicated that the robot did not interfere with the wearer motion during manual material handling. The responses for Q5 and Q6 revealed that the variable magnitude of assistive torque was sufficient and effectively transmitted to the human body. The response for Q7 indicated a positive feedback regarding practical usability.

## 4. Discussion

Ideally, the magnitude of the assistive torque of the modified CVGC-II should be identical in the UP and DOWN phases at each interval angle during torque profile measurement. However, hysteresis was observed in the UP phase. In this case, the reason for the hysteresis was the increased friction between internal parts such as the spring follower and lever, because of the increased spring constant relative to that of the CVGC-II. Nevertheless, the modified CVGC-II successfully increased the maximum value and variable range of assistive torque to provide proper back support.

The evaluation of the assistive torque transmission performance of the robot structure confirmed that the assistive torque was properly transmitted to the human body in the DOWN phase. However, a magnitude difference was observed between the original torque of the modified CVGC-II and transmitted torque through the robot structure. Identifying the exact cause of this difference requires further analysis, but we assume that the reason for the difference could be an elastic deformation of the structure. The carbon pipes and 3D-printed parts, such as ABS, can be easily deformed elastically. The output torque of the modified CVGC-II led to not only the compression of the thigh cuff but also the deformation of elastic components. In the UP phase, the magnitude of the assistive torque was lower than that in the DOWN phase because of the hysteresis of the assistive torque source device. Despite these differences caused by elastic deformation, the experiment successfully evaluated the variable assistive torque transmitting performance of the structure of AeBS.

The hysteresis of modified CVGC-II that occurred in UP phase is the limitation of the current study. The hysteresis makes it impossible to provide stable and even assistive torque throughout the lifting and lowering phase. This means that the wearer cannot be provided full assistive torque while lifting material up. As a result, the hysteresis may reduce the effectiveness of the robot in preventing low back injuries. Thus, the hysteresis problem should be resolved by reducing the internal friction in further research.

In the usability evaluation, the responses for Q1, Q2, and Q7 were lower than those for Q3 to Q6. We assume that the reason for the response could be the increased effective mass of both legs. Because the main frame of the CVGC-II module was connected to the leg to use the relative rotation between the upper body and leg, the effective mass and moment of inertia of the thigh potentially increased. The increased effective mass and moment of inertia likely affected the perceived weight while wearing the robot and moving the legs.

The energy efficiency of the AeBS was relatively high compared to previously developed quasi-passive exoskeletons. Moreover, the energy consumption of 1 W used to adjust the magnitude of assistive torque was less than the energy consumption of MRLift [25], which is 5 W. Additionally, the AeBS improved user comfort. Through the spine collision-avoiding connecting rod, the AeBS corresponded to spine motion, whereas MRLift has no structure for spine curvature. Through thigh cuff rotation, the AeBS ensured secure contact of the thigh cuff, which is not the case with MRLift and another quasi-passive exoskeleton prototype [23].

Overall, this study was aimed at developing a quasi-passive back-support exoskeleton capable of adjusting the magnitude of assistive torque without range-of-motion restriction while consuming low energy. The performance of the variable assist was evaluated using test benches. The evaluation confirmed that variable assistance could be effectively achieved throughout the assistive torque source device and transmitted through the robot structure. However, it also confirmed that the hysteresis of the assistive torque source device occurred and affected the assist performance of the robot. Nevertheless, AeBS can mitigate the limitations of the existing quasi-passive back-support exoskeletons by using a lever mechanism with a variable pivot and screw mechanism. Therefore, addressing the hysteresis problem of modified CVGC-II can further enhance the variable assist performance regardless of the lifting phase condition.

## 5. Conclusions

In this paper, we proposed a quasi-passive exoskeleton designed to provide variable assistive torque without restricting the range-of-motion while operating in an energy-efficient way. To evaluate the variable assist function of the robot, we measured the variable assistive torque of the modified CVGC-II and validated the torque transmitting performance of the robot structure. Through evaluation, AeBS mitigates the restriction of the range-of-motion and energy efficiency problems caused by the change of assistive torque of existing quasi-passive exoskeletons. Additionally, we verified a questionnaire survey to examine whether the variable assistive torque could be effectively provided to the human body and if the robot structure and joints cause any pain or discomfort while using the robot. Through qualitative evaluation, the human-inspired robot joint and ensuring free motion functions work well and contribute to applying quasi-passive exoskeleton robots in actual industrial sites. Currently, we are developing an improved version of AeBS with less hysteresis and conducting human-subject evaluations involving manual material handling tasks to evaluate whether the robot can reduce low back pain.

## Figures and Tables

**Table 1:** The comparison of the weight of the active, passive and quasi-passive back-support exoskeletons.

Device Name	Actuation Class	Actuation Technology	Mass (kg)
Robo-Mate [19]	Active	Electrical motor	11
XoTrunk [13]	Active	Electrical motor	8
H-WEX [12]	Active	Electrical motor	4.5
BackX [10]	Passive	Gas spring	3.4
Laevo [21]	Passive	Gas spring	2.3
Spexor [15,16]	Passive	Coil spring + carbon beam	6.7
MRLift [25]	Quasi-passive	Coil spring + MR Fluid	3.95
AeBS	Quasi-passive	CoiLeaf spring + micromotor	5

**Table 2:** Question texts and scores for the usability questionnaire.

Index	Question Text (1 = Strongly Disagree, 2 = Disagree, 3 = Neutral, 4 = Agree, 5 = Strongly Agree)	Mean ± SD
Q1	Did you feel robot is lightweight?	3.36 ± 0.79
Q2	Did you feel uninterrupted while wearing robot?	3.39 ± 1.08
Q3	Did you feel comfortable while lifting/lowering tasks wearing robot?	4.16 ± 0.61
Q4	Did you feel no pain while lifting/lowering tasks wearing robot?	4.36 ± 0.60
Q5	Did you feel assistance by robot?	4.26 ± 0.62
Q6	Did you feel difference between adjustable assist?	4.14 ± 0.64
Q7	Do you think that robot can be helpful in real work environment?	3.98 ± 0.71

## Data Availability

Data are contained within the article and supplementary materials.

## Supplementary Materials

The following supporting information can be downloaded at: https://www.mdpi.com/article/10.3390/biomimetics9030173/s1, Video S1: Development of Quasi-Passive Back-Support Exoskeleton with Compact Variable Gravity Compensation Module and Bio-Inspired Hip Joint Mechanism.

## References

[1] Bracko M.R. (2004). Can We Prevent Back Injuries?. ACSM’s Health Fit. J..

[2] McGill S.M. (1997). The biomechanics of low back injury: Implications on current practice in industry and the clinic. J. Biomech..

[3] Dolan P., Adams M.A. (1998). Repetitive lifting tasks fatigue the back muscles and increase the bending moment acting on the lumbar spine. J. Biomech..

[4] Panjabi M.M. (2006). A hypothesis of chronic back pain: Ligament subfailure injuries lead to muscle control dysfunction. Eur. Spine J..

[5] Dagenais S., Caro J., Haldeman S. (2008). A systematic review of low back pain cost of illness studies in the United States and internationally. Spine J..

[6] Katz J.N. (2006). Lumbar Disc Disorders and Low-Back Pain: Socioeconomic Factors and Consequences. JBJS.

[7] Hartvigsen J., Hancock M.J., Kongsted A., Louw Q., Ferreira M.L., Genevay S., Hoy D., Karppinen J., Pransky G., Sieper J. (2018). What low back pain is and why we need to pay attention. Lancet.

[8] Yu H., Choi I.S., Han K.-L., Choi J.Y., Chung G., Suh J. (2015). Development of a Stand-alone Powered Exoskeleton Robot Suit in Steel Manufacturing. ISIJ Int..

[9] Toxiri S., Calanca A., Ortiz J., Fiorini P., Caldwell D.G. (2018). A Parallel-Elastic Actuator for a Torque-Controlled Back-Support Exoskeleton. IEEE Robot. Autom. Lett..

[10] Kazerooni H., Tung W., Pillai M. Evaluation of Trunk-Supporting Exoskeleton. Proceedings of the Human Factors and Ergonomics Society Annual Meeting.

[11] Wehner M., Rempel D., Kazerooni H. Lower Extremity Exoskeleton Reduces Back Forces in Lifting. Proceedings of the ASME 2009 Dynamic Systems and Control Conference.

[12] Ko H.K., Lee S.W., Koo D.H., Lee I., Hyun D.J. (2018). Waist-assistive exoskeleton powered by a singular actuation mechanism for prevention of back-injury. Robot. Auton. Syst..

[13] Poliero T., Fanti V., Sposito M., Caldwell D.G., Natali C.D. (2022). Active and Passive Back-Support Exoskeletons: A Comparison in Static and Dynamic Tasks. IEEE Robot. Autom. Lett..

[14] Bosch T., van Eck J., Knitel K., de Looze M. (2016). The effects of a passive exoskeleton on muscle activity, discomfort and endurance time in forward bending work. Appl. Ergon..

[15] Näf M.B., Koopman A.S., Baltrusch S., Rodriguez-Guerrero C., Vanderborght B., Lefeber D. (2018). Passive Back Support Exoskeleton Improves Range of Motion Using Flexible Beams. Front. Robot. AI.

[16] Baltrusch S.J., van Dieën J.H., Koopman A.S., Näf M.B., Rodriguez-Guerrero C., Babič J., Houdijk H. (2020). SPEXOR passive spinal exoskeleton decreases metabolic cost during symmetric repetitive lifting. Eur. J. Appl. Physiol..

[17] Schipplein O.D., Trafimow J.H., Andersson G.B.J., Andriacchi T.P. (1990). Relationship between moments at the L5/S1 level, hip and knee joint when lifting. J. Biomech..

[18] Hwang S., Kim Y., Kim Y. (2009). Lower extremity joint kinetics and lumbar curvature during squat and stoop lifting. BMC Musculoskelet. Disord..

[19] Toxiri S., Näf M.B., Lazzaroni M., Fernández J., Sposito M., Poliero T., Monica L., Anastasi S., Caldwell D.G., Ortiz J. (2019). Back-Support Exoskeletons for Occupational Use: An Overview of Technological Advances and Trends. IISE Trans. Occup. Ergon. Hum. Factors.

[20] Baltrusch S.J., van Dieën J.H., Bruijn S.M., Koopman A.S., van Bennekom C.A.M., Houdijk H. (2019). The effect of a passive trunk exoskeleton on metabolic costs during lifting and walking. Ergonomics.

[21] Koopman A.S., Kingma I., de Looze M.P., van Dieën J.H. (2020). Effects of a passive back exoskeleton on the mechanical loading of the low-back during symmetric lifting. J. Biomech..

[22] Jamšek M., Petrič T., Babič J. (2020). Gaussian Mixture Models for Control of Quasi-Passive Spinal Exoskeletons. Sensors.

[23] Callens T., Ducastel V., De Schutter J., Aertbeliën E. (2023). Using Intent Estimation and Decision Theory to Support Lifting Motions with a Quasi-Passive Hip Exoskeleton. arXiv.

[24] Hassan M., Kennard M., Yagi K., Kadone H., Mochiyama H., Suzuki K. MRLift: A Semi-active Lower Back Support Exoskeleton based on MR Fluid and Force Retention Technology. Proceedings of the 2019 IEEE/RSJ International Conference on Intelligent Robots and Systems (IROS).

[25] Kennard M., Yagi K., Hassan M., Kadone H., Mochiyama H., Suzuki K. (2023). Variable-Damper Control Using MR Fluid for Lower Back Support Exoskeleton. IEEE/ASME Trans. Mechatron..

[26] Kim J., Moon J., Ryu J., Lee G. (2022). CVGC-II: A New Version of a Compact Variable Gravity Compensator With a Wider Range of Variable Torque and Energy-Free Variable Mechanism. IEEE/ASME Trans. Mechatron..

[27] Moon J., Ryu J., Kim J., Lee G. (2022). CoiLeaf spring: A hybrid system of coil and leaf springs for maximizing space utilization and energy storage. Mech. Mach. Theory.

[28] Butler R.J., Plisky P.J., Southers C., Scoma C., Kiesel K.B. (2010). Biomechanical analysis of the different classifications of the Functional Movement Screen deep squat test. Sports Biomech..

[29] Caldwell D.G., De Momi E., Di Natali C., Ortiz J., Sposito M., Toxiri S. (2020). Exoskeleton kinematic design robustness: An assessment method to account for human variability. Wearable Technol..

[30] Muramatsu Y., Kobayashi H., Sato Y., Jiaou H., Hashimoto T., Kobayashi H. (2011). Quantitative Performance Analysis of Exoskeleton Augmenting Devices-Muscle Suit-for Manual Worker. Int. J. Autom. Technol..

